# MAPPING LONGEVITY PATHWAYS THROUGH MITOCHONDRIAL DYSFUNCTION: ROLES OF THE MICROBIOME AND METABOLISM IN AGING

**DOI:** 10.1093/geroni/igae098.2627

**Published:** 2024-12-31

**Authors:** Alessandro Bitto

**Affiliations:** University of Washington, Seattle, Washington, United States

## Abstract

Biological aging is characterized by a progressive deterioration of fundamental biological functions in living organisms, often codified as hallmarks or pillars. Longevity interventions impact these hallmarks, slowing down or even reversing their deterioration. Thus, we hypothesize that longevity interventions may be applied to chronic diseases characterized by failure of one or more of these fundamental biological processes, regardless of biological age. Herein, we describe how geroscience can be leveraged to gather new insight into etiology, progression, management, and treatment of chronic mitochondrial disease. With the aid of a mitochondrial dysfunction model, we investigate the effects on intermediate metabolism and the intestinal flora of two longevity interventions, the antidiabetic drug acarbose and the mTOR inhibitor rapamycin. Furthermore, we propose that models of chronic disease can be leveraged to study the mechanisms of action of known longevity pathways and develop new interventions. We describe the critical role of fatty acid metabolism in the treatment of mitochondrial disease and hypothesize that similar mechanisms may contribute to the longevity effects of mTOR inhibition in aging models.

